# Predation increases multiple components of microbial diversity in activated sludge communities

**DOI:** 10.1038/s41396-021-01145-z

**Published:** 2021-12-01

**Authors:** Alfred Burian, Daisy Pinn, Ignacio Peralta-Maraver, Michael Sweet, Quentin Mauvisseau, Ozge Eyice, Mark Bulling, Till Röthig, Pavel Kratina

**Affiliations:** 1 Aquatic Research Facility, Environmental Sustainability Research Centre, University of Derby, Derby, UK; 2 Marine Ecology Department, Lurio University, Nampula, Mozambique; 3 Department of Computational Landscape Ecology, UFZ– Helmholtz Centre for Environmental Research, Leipzig, Germany; 4 School of Biological and Behavioural Sciences, Queen Mary University of London, London, UK; 5 Thames Water Utilities Ltd, Reading, UK; 6 Department of Ecology, University of Granada, Granada, Spain; 7 Research Unit Modeling Nature (MNat), University of Granada, Granada, Spain; 8 Natural History Museum, University of Oslo, Oslo, Norway; 9 Department of Bioresources, Fraunhofer Institute for Molecular Biology and Applied Ecology, Giessen, Germany

**Keywords:** Microbial communities, Biodiversity

## Abstract

Protozoan predators form an essential component of activated sludge communities that is tightly linked to wastewater treatment efficiency. Nonetheless, very little is known how protozoan predation is channelled via bacterial communities to affect ecosystem functioning. Therefore, we experimentally manipulated protozoan predation pressure in activated-sludge communities to determine its impacts on microbial diversity, composition and putative functionality. Different components of bacterial diversity such as taxa richness, evenness, genetic diversity and beta diversity all responded strongly and positively to high protozoan predation pressure. These responses were non-linear and levelled off at higher levels of predation pressure, supporting predictions of hump-shaped relationships between predation pressure and prey diversity. In contrast to predation intensity, the impact of predator diversity had both positive (taxa richness) and negative (evenness and phylogenetic distinctiveness) effects on bacterial diversity. Furthermore, predation shaped the structure of bacterial communities. Reduction in top-down control negatively affected the majority of taxa that are generally associated with increased treatment efficiency, compromising particularly the potential for nitrogen removal. Consequently, our findings highlight responses of bacterial diversity and community composition as two distinct mechanisms linking protozoan predation with ecosystem functioning in activated sludge communities.

## Introduction

The treatment of wastewater using activated sludge communities represents arguably the largest single biotechnological process world-wide [1]. This crucial ecosystem service is provided by diverse communities of bacteria, protozoans and metazoan grazers [2–5]. Past research has highlighted that the effective biological treatment of wastewater critically depends on the composition and diversity of bacterial assemblages [6, 7]. However, also protozoan predators play a key role in maintaining treatment efficiency in activated sludge [8–11]. Characteristic predators, such as ciliates and heterotrophic nanoflagellates (HNFs) express dynamic changes in their densities and complex successional patterns [12, 13]. Their total density is, nonetheless, often positively associated with essential bacterial functions, such as denitrification and the reduction of biological oxygen demand (BOD) in treatment plant effluent [9].

The positive impacts of protozoan predation on ecosystem functioning have been traditionally explained by stimulating effects on bacterial physiology [8, 10]. For example, protozoa may excrete growth-stimulating substances that boost bacterial activity [4]. Predation plays also an important role maintaining high bacterial growth rates enhancing nutrient re-mineralisation and carbon respiration [10, 14, 15]. In contrast, direct impacts of predation on prey community composition are much less studied in activated sludge communities [16, 17]. However, the strength of direct predator–prey interactions [18] and their importance for ecosystem functioning is well demonstrated in other systems [16, 19, 20], highlighting a potential route for further optimisations of biological wastewater treatments.

One link with potentially considerable consequences for ecosystem functioning is the relationship between protozoan predation and bacterial diversity. Diversity is well-known to increase the rate of ecosystem functioning [21–23] and promote multiple aspects of ecosystem stability [24, 25], including a greater toxin resistance of more diverse activated sludge communities [26]. However, the relationship between predation pressure and prey diversity is not always positive [27, 28], and both positive and negative effects of predation on prey diversity have been documented [29–31]. This has led to the postulation of a hump-shaped relationship between prey diversity and the strength of predation pressure [27, 32].

**Fig. 1:**
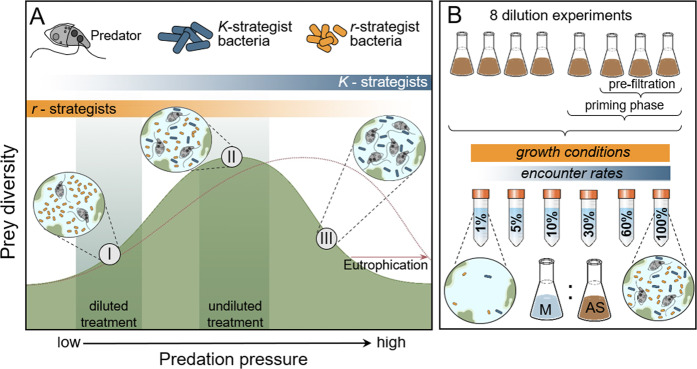
The postulated hump-shaped relationship between predation pressure and prey diversity and an overview of the studies experimental set-up. **A** At low pressure (I), predators are unable to control rapidly growing *r*-strategists resulting in the exclusion of slower growing prey taxa [27]. At intermediate predation pressure (II) more *K*-strategists resistant to predation start to emerge and the co-existence of different strategies leads to a peak in prey diversity. A further increase in predation pressure (III) benefits *K*-strategists as it promotes the exclusion of less defended, opportunistic prey. This relationship is proposed to be mediated by ecosystem productivity [i.e. nutrient level, [28, 33]]. In extremely nutrient rich water treatment reactors (dotted line), this is expected to lead to a largely positive impact of protozoan predation on bacterial diversity. We performed eight experiments (**B**) in which we manipulated predation pressure by diluting activated sludge (AS) communities with growth media (M). The dilutions resulted in a reduction in predator-prey encounter rates and hence predation pressure, while growth conditions remained relatively constant, effectively shifting conditions to the left on the x-axis in panel A. Four out of 8 experiments where pre-conditioned in chemostats and three of them were pre-filtered to remove the largest fraction of predators from experimental communities and to diversify the types of communities tested.

This hump-shaped relationship is thought to emerge because intermediate predation pressure facilitates the co-existence of multiple prey strategies [28, 33]. More predation resistant *K*- and opportunistic *r*-strategists may equally persist at intermediate levels of top-down control (Fig. 1A). Predator-mediated prey co-existence is particularly favoured in systems where predator densities fluctuate over time [34], as frequently observed in activated sludge communities [4, 35]. The strength of predation pressure that maintains such peak prey diversity is believed to be mediated by nutrient concentrations and resulting ecosystem productivity [27]. Higher productivity is reflected in higher prey population growth rates, which requires a stronger top-down control of opportunistic *r*-strategists to facilitate prey coexistence (Fig. 1A). Activated sludge reactors are engineered ecosystems characterised by high nutrient concentrations and microbial carrying capacities [e.g. [8]]. The predation pressure required to maintain peak prey diversity is therefore expected to be much higher than in many natural ecosystems, potentially resulting in almost linear relationships between predation pressure and prey diversity (Fig. 1A). This conceptual framework may thus explain the frequently observed positive knock-on effects of predator density on treatment efficiency in activated sludge communities [9, 10].

In addition to impacting prey diversity, protozoans can alter the identity of dominant bacterial taxa [17, 36] and selective predation may change the relative densities of functionally important bacteria in water treatment reactors. Indeed, different protozoans such as bacterivorous *Chilodonella* and *Colpidium* are associated with higher treatment efficiency [37], whereas others (e.g. the HNFs *Bodo* and *Polytoma*) appear to have predominantly negative impacts [9]. Currently, the mechanisms that underlie such shifts in functional identity and the direct impacts of protozoan predation on bacterial community composition remain unexplored. Moreover, the relationship between prey and predator diversity is conceptually poorly understood [38, 39], limiting our potential to further optimise sewage treatment by activated sludge communities.

Our aim was to determine the effect of protozoan predation intensity on bacterial diversity and community composition in activated sludge. We used a series of dilution experiments, developed to quantify the impacts of predation pressure on plankton communities [40, 41], in order to experimentally control the strength of protozoan predation. Metabarcoding and flow cytometric analyses of prey and predators allowed us to characterise microbial communities and responses to reductions in top-down control. Specifically, we quantified changes in bacterial alpha and beta diversity in response to reduced levels of predation pressure. Furthermore, we investigated relationships between bacterial and protozoan diversity to evaluate inter-trophic linkages in richness, evenness and genetic diversity. Finally, we examined whether reduced top-down control resulted in systematic shifts in community composition, gauging potential consequences for the efficiency of wastewater treatment plants.

## Materials and methods

### Sample collection and preparation

Activated sludge samples were collected from the Severn Trent wastewater treatment plant in Derby (UK) between 9:30 and 11:30 am on 14th February 2019. Aeration tanks contained four fully separated lanes (no water exchange). We collected 800 mL of suspended activated sludge from each of the four lanes as inocula for laboratory experiments. We also collected 40 L of influent to the biological treatment tank, i.e. wastewater that had already undergone primary treatment. These 40 L were filtered on site (75 uL mesh sieves to remove debris), autoclaved and used for the preparation of experimental growth media. All samples were stored in insulated coolers, kept in the dark and transported to the laboratory within 3 h.

### Priming of communities prior to experiments

In total, we conducted eight dilution experiments (Fig. 1B). Four of these experiments (labelled as experiments 1–4) were directly inoculated with microbial communities from one of the four treatment plant lanes (all four lanes at Derby Treatment plant were sampled). The other four experiments (experiments 5–8) were established from the outflow of four different continuous flow-through chemostats, which were inoculated with activated sludge (the same sample from lane 1; see Fig. S1 for details about chemostat design and operation). Chemostat were run for two weeks before the start of dilution experiments and they were implemented for two reasons as conditioning pre-treatments for microbial communities. First, activated sludge community composition can be substantially influenced by bacteria entering over the inflow [42]. The experiments with cultures from chemostats that used filtered and sterilised media, marginalised the impact of inflow bacteria and allowed to control for potentially confounding effects on community composition. Second, the use of chemostats allowed to diversify experimental communities, which allowed us to double the number of experiments and increase the generality of our findings. Dilution rates in chemostats impose unselective background mortality rates on predator and prey taxa and filtration of inocula selectively excludes certain community members (e.g. rotifers and larger, tentatively carnivorous ciliates). We therefore initiated chemostats with either unfiltered or prefiltered (50 µm mesh size) activated sludge samples, and operated chemostats at different dilution rates in order to prime different predator assemblages (chemostat for experiment 5: unfiltered and a dilution rate of 0.35 d^−1^; chemostats for experiments 6-8: pre-filtered with dilutions rates of 0.35, 0.5, 0.2 d^-1^, respectively). The use of autoclaved treatment plant influent, which is rich in organic substrates [43], as growth media helped to maintain a high microbial diversity over the course of the conditioning phase (Fig. S2).

### Experimental set-up and sampling

Dilution experiments are based on the principle of diluting microbial communities with organism free ambient water [40]. The impact of predation on prey community composition and diversity can be assessed by this method because predation pressure is reduced (lowered encounter rates), whereas growth conditions for prey species are relatively unaffected [40]. For each of our eight experiments, we established six duplicated dilution treatments in 50 mL falcon tubes (in total 96 microcosms with 5 mL volume). Microcosms were established by combining an inoculum with autoclaved and filtered (0.2 µm nylon filters) influent. The six dilution treatments per experiment included 100%, 60%, 30%, 10%, 5% and 1% of inoculum. Experiment 4 was inadvertently set up with a slightly altered dilution series including 100%, 38%, 24%, 10%, 6.6%, 2.4% of inoculum. To obtain enough DNA for next-generation sequencing, additional microcosms for the 100% and 1% inoculum treatments were set up containing larger volumes (20 mL and 200 mL total volume, respectively; two replicates each). Microcosms were continuously homogenised on a shaking table (120 rotations min^−1^) and kept in the dark at 20 ± 0.5 ˚C. After 24 h, all microcosms were sampled for flow cytometry and the lowest and highest dilution were sampled for next-generation sequencing. Prior to the experiment, all inocula were also sampled in triplicates to determine starting conditions.

For flow cytometry, 0.9 mL from each microcosm were sampled to measure ratios of high nucleic acid (HNA) to low nucleic acid (LNA) bacterial cells, and 2.7 mL were taken to enumerate HNF densities. Samples were fixed with paraformaldehyde and glutaraldehyde, shock frozen in liquid nitrogen and stored at −80 ˚C following protocols by Gasol and Morán [Fig. S3; [44]]. Samples for DNA extraction were collected by pressure filtration and material was collated until filters clogged (20 mL from undiluted communities, 100 mL from diluted communities; 0.2 µm polycarbonate filters, Cyclopore Whatman, UK). All filters were shock frozen and stored at −80 ˚C.

### Flow cytometry and high-throughput sequencing

In all experiments, we assessed prey and predator community composition applying a meta-barcoding approach. Additionally, we used flow cytometry to evaluate HNA–LNA ratios of bacteria, which are interpreted as a potential indicator of bacterial cell activity [45]. Enumeration of bacterial density with flow cytometry was not reliable as many taxa were particle-associated confounding accurate quantification. Moreover, we quantified HNF densities in undiluted samples (deemed technically not feasible in undiluted samples), representing one important fraction of grazer communities.

HNF densities and HNA–LNA bacteria ratios were analysed on a BD Accuri C6 automatic flow cytometer (BD Biosciences, USA) following largely the protocol by Gasol and Morán [[44]; for further details see SI, section S1]. DNA for meta-barcoding analyses was extracted with the QIAGEN DNeasy Blood and Tissue Kit, following the manufacturer’s protocols. The 16S rRNA gene (V3-V4 region) from the DNA samples were amplified using the universal bacterial primers [46], 515F (5′-GTGYCAGCMGCCGCGGTAA-3′) and 806R (5′-GGACTACNVGGGTWTCTAAT-3′). Additionally, we targeted eukaryotic sequences amplifying the 18S rRNA gene using the primers 574*f (5′-CGGTAAYTCCAGCTCYV-3′) + 1132r - (5′-CCGTCAATTHCTTYAART-3′) based on Hugerth et al. [47]. Barcodes were added via PCR and the amplicons were then cleaned up using a bead-based kit (AMPure XP, Beckman Coulter, US), pooled and sequenced (2 × 250 bp) on the MiSeq (Illumina, US) platform [48].

### Sequence and statistical analysis

Raw sequence reads were first quality controlled for chimera and sequence fragments (72% and 64% of raw sequences remained for prokaryotes and protozoa respectively) in QIIME2 [49]. DNA-polymerase sequencing errors were accounted for using the dada2 algorithm [50] to attain relative frequencies of amplicon sequence variants (ASVs). The mean number of reads per sample was 69,018 ± 12,345 (SD) for prokaryotes and 29,561 ± 18,502 for protozoa. Total number of reads in some protozoan samples were relatively low due to primer or PCR inhibition. We eliminated samples with low total copy number (<15,000) from further analysis before rarefication, resulting in a replication of 10, 12 and 15 samples from the reduced grazing, the ambient grazing and start samples, respectively. The taxonomic identity of prokaryote ASVs was determined using the SILVA RNA database at 99% similarity [release 138; [51]] and a multinomial Naive Bayes classifier trained for the selected V4 sequence in QIIME 2. However, we maintained the recently challenged family of the Comamonadaceae to aid comparability with earlier studies. All non-assigned ASVs at the Kingdom level, and all chloroplast ASVs, were removed from the analyses. As bacteria dominated our samples (only 0.12% of ASVs were Archea), we henceforth refer to prokaryote as ‘bacterial’ ASVs. Taxonomic identity of numerically important ASVs was confirmed by blast-searching and checking manually the 100 most abundant ASVs across all samples on the NCBI database. Protozoan sequences were analogously classified using the SILVA database at 99% similarity [51]. To assure that we only considered bacterial predators and avoided contamination (e.g. mammalian DNA), we considered only taxa that were affiliated to the classes Alveolata, Rhizaria, Discoba, Discosea or Holozoa. Within Holozoa, we also included the potentially bacterivorous taxa Chromadorea, Bdelloidea and Phyllopoda. However, as Holozoa comprised only a small subfraction of all taxa and reads, we refer hereafter to ‘predator ASVs’ as protozoans. Phylogenetic trees were constructed using the FastTree software [52]. All samples were uploaded to NCBI database (PRJNA726629).

The effect of dilution on alpha diversity was assessed by comparing ASV richness, ASV evenness (Pielou’s evenness) and genetic diversity measured by the Faith index [53], after rarefaction to standardise sampling effort to the lowest sequencing depth. We also assessed mean phylogenetic distinctiveness of ASVs following Tsirogiannis and Sandel [54]. Phylogenetic distinctiveness is a measure based on the Faith index, which removes the effect of species richness on genetic diversity using a bootstrapping approach (1000 iterations). We applied a linear mixed effects model to determine differences in diversity metrics among communities at the start of incubations as well as in diluted and undiluted communities (also referred to as reduced-predation and predation treatment, respectively) at the end of incubations. Experiment identity (experiment 1–8) was accounted for as random effect. We also compared relative abundances of ASVs between predation and reduced predation treatments at the end of the experiments using a non-parametric factorial analysis after Wobbrock et al. [55], again including experiment number as random effect.

A community similarity matrix was established based on Bray–Curtis similarity and visualised using non-metric multidimensional scaling (NMDS; stress value of 0.08). We then applied ANOVA with subsequent Tukey post-hoc tests to evaluate whether (i) communities in the predation or reduced-predation treatments at the end of the experiments were more similar in composition to the starting (inocula) communities and (ii) beta diversity (i.e. dissimilarity among communities) was different among the communities in the start inocula, predation or reduced predation treatments. Non-parametric tests were used when variance-homogeneity could not be achieved through transformation. Finally, we used ordinary least squares regressions to test the effect of HNF densities on prey alpha diversity within treatments (i.e. a separate analysis for communities with reduced and normal predation pressure) to assess whether this relationship is consistent at low and high predation pressure. Because we were able to measure HNF densities in undiluted samples only, we used the starting HNF densities for these within treatment assessments. We examined whether regression model residuals met the assumptions of normality, equal variances, and were not autocorrelated. All implemented regression models met these requirements. Nonlinearity between dependent and explanatory variables was assessed visually and by comparing models with log-transformed, exponentially-transformed and untransformed independent variables based on the smallest Akaike’s Information Criterion [AIC, corrected for small sample size; [56]].

Finally, we applied two complementary approaches to examine how shifts in bacterial community composition affected their putative functionality. First, we used an automated, taxonomy inferred approach to predict potential functional differences between treatments [METAGENassist [57]; results only presented in SI]. Second, we related our results to a global meta-analysis of activated sludge communities [7], which provides the functional association of commonly occurring taxa (>20% occurrence across samples in meta-analysis). We compared all ASVs related to those taxa and evaluated significant responses in relative abundance to microcosm dilution. All analyses were performed in R, version 3.6 [58], and all R-scripts are provided in Annex 1.

## Results

**Fig. 2:**
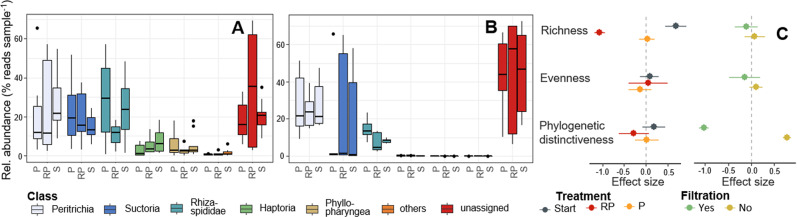
Protozoan community composition and determinants of their biodiversity. Protozoan community differed between unfiltered (**A**) and pre-filtered (**B**) communities. In each panel, box plots for each taxonomic class in microcosms with ambient predation pressure (P), reduced predation pressure (RP) and at starting conditions (S) are illustrated. In **C**, responses of protozoan diversity (i.e. taxa richness, evenness and phylogenetic distinctiveness) to treatment implementation and filtration in the priming phase of the experiment (50 μm) are displayed. Points represent sample means, bars represent ±1 standard error of the mean.

Experimental predator communities had a mean ASV-richness of 72 ± 28 (SD) and were dominated in richness and relative abundance by ciliates (mainly Peritrichia and Suctoria) and amoeba (primarily Rhizaria; Fig. 2, Fig. S4). Both treatment implementation (i.e. dilution to reduce prey encounter rates and thus predation pressure) and filtration, during the experimental conditioning phase, had significant impacts on predator diversity (Tables 1 and TS1). However, they affected different components of predator diversity. Whereas filtration significantly reduced taxa richness, dilution lowered phylogenetic diversity of predators (Fig. 2C, Table S1). Filtration during the conditioning phase also had a marked impact on predator community composition, significantly reducing relative densities of Haptoria, Phyllopharyngea and other rare protozoan families (paired Wilcox-Test, *W* > 326, *p* < 0.001). Yet, overall protozoan taxonomy was not well resolved as 31.1% of ASVs could only be assigned to class level.

**Table 1:** The effects of reduced predation pressure on ASVs associated with the globally most common bacterial taxa in activated sludge communities.

Taxa	Functionality after Wu et al. [7]	Comments	Increase	Decrease	Total change [%]	*p* value taxa level
*Arcobacter*	BOD (+), COD (++), NH_4_ (−)	Facultative anaerobic, diverse group that includes photogenes	0/62	0/62	+93	0.33
*Candidatus* Accumulibacter	COD (++)	Known as PAO, may increase TP removal	0/15	2/15	−42	0.07
Chitinophagaceae	BOD (++), COD (++), NH_4_ (++), TP (++)	Degradation of cellulose and chitin	0/379	1/379	−57	0.001
*Cloacibacterium*	BOD (++), NH_4_ (−)		0/10	0/10	+14	0.07
Comamonadaceae (excl. *Rhodoferax*)	BOD (++), COD (++), NH_4_ (+), TP (++)	Important for denitrification	1/64	4/64	−60	0.008
*Dokdonella*	NH_4_ (+)		0/20	2/20	−68	0.001
*Haliangium*	COD (+), TP (+)	Chemoautotrophs	0/169	3/169	−36	0.02
*Nitrospira*	TP (−)	Nitrite and hydrogen oxidiser, potential AOB	0/16	4/16	−45	0.001
Moraxcellaceae (inc. *Acinetobacter*)	BOD (+), COD (++), TP (+)	Support aggregate for-mation and P removal	18/416	0/416	+1026	0.001
Rhodocyclaceae (excl. *Zooglea*, *Can*. Accumulibacter)	COD (++), TP (−−)		4/192	6/192	+5	0.83
*Rhodoferax*	BOD (++), COD (+), NH_4_ (+), TP (++)	anoxygenic photo-organotrophy de-grading C-compounds as C-sources	0/5	1/5	−51	0.002
Saprospiraceae	BOD (++), NH_4_ (+), TN (++), TP (+)	Protein-hydrolysing bacteria, but may also support bulking	0/384	23/384	−75	0.001
*Sulfuritalea*	BOD (−−), NH_4_ (−)	Denitrifying bacteria	0/27	2/27	−66	0.001
*Turneriella*	COD (++)	Degradation of fats	0/29	0/29	−19	0.23
Xanthomonadaceae	BOD (+), NH_4_ (++)	Support sludge granulation	3/192	2/192	+158	0.05
*Zoogloea*	BOD (++), COD (++), NH_4_ (+), TN (+), TP (+)	Denitrifies, degrading benzonatate rings	0/93	1/93	−5	0.34
*Zymomonas*	BOD (−−), COD (−), NH_4_ (−−), TN (−), TP (−−)	Alcohol production	−	−	−	−

Displayed are the most common taxa and their impacts on wastewater treatment efficiency according to Wu et al. [7]. The numbers of ASV associated with these taxa illustrate either an increase or a decrease of relative densities in microcosms with reduced predation pressure. Numbers behind the slash denote the total recorded ASVs. Beneficial ecosystem functions include removal of biological oxygen demand (BOD), chemical carbon demand (COD), ammonium (NH_4_), total nitrogen (TN) and total phosphorus (TP) from effluent. Two signs (either + or –) indicate highly significant effects (*p* < 0.01), one sign indicates significant association with a certain function (*p* < 0.05). PAO represents polyphosphate-accumulating organisms and AOB represents ammonia-oxidising bacteria.

**Fig. 3:**
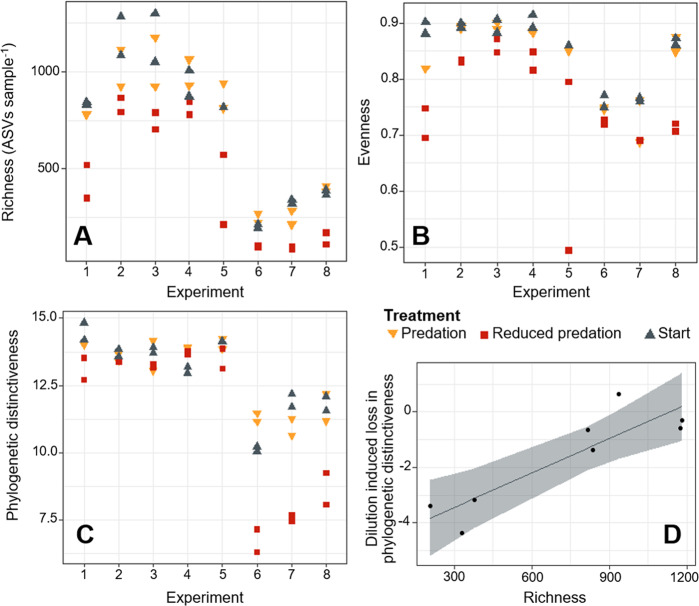
Prey biodiversity responses to changes in predator communities. Prokaryotic ASV richness (**A**), evenness (**B**) and phylogenetic distinctiveness (**C**) decreased in the diluted treatment (red) compared starting conditions (blue and the undiluted trated (yellow). Results for each of the 8 experiments are plotted separately to account for systematic differences in starting conditions across experiments. **D** The decrease in phylogenetic distinctiveness in the treatments with reduced predation was positively related to the starting ASV richness of experiments (linear regression; *R*^2^ = 0.75, *p* = 0.03, *y* = 0.004*x* − 4.7). Grey line denotes the predicted relationship and the shaded grey area represents the 95% confidence interval of the slope.

The diversity of bacterial prey communities was strongly influenced by the experimental dilution and filtration during the conditioning phase (Fig. 3). Both manipulations additively reduced different bacterial diversity components, including richness (*R*^2^ = 0.82, *p* < 0.001), evenness (*R*^2^ = 0.56, *p* < 0.001) and phylogenetic distinctiveness (*R*^2^ = 0.55, *p* < 0.001). Notably, communities with high richness were less sensitive to negative effects of dilution highlighted by their lower loss rates in phylogenetic distinctiveness in diluted microcosms (*p* = 0.003, *R*^2^ = 0.75, *y* = 0.004*x* -4.7; Fig. 2D). Prey diversity was also linked to the diversity of protozoan predators (Table S2), although predator diversity impacts were additive to and not underlying filtration and dilution effects. Further, the impact of predator diversity was variable in effect direction and neither consistently negative nor positive. E.g., bacterial phylogenetic distinctiveness was affected positively by protozoan richness, but negatively by protozoan evenness and phylogenetic distinctiveness of predators. Protozoan phylogenetic distinctiveness also had a weak but significant negative effect on bacterial evenness.

**Fig. 4:**
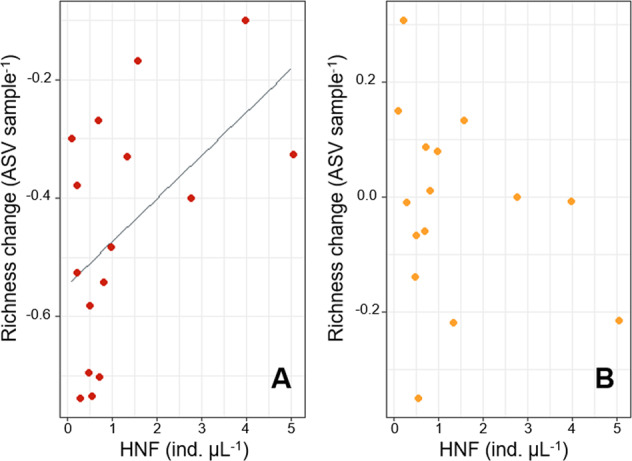
Association between heterotrophic nanoflagellates (HNF) and bacterial diversity. HNF were positively associated with changes in taxa richness over the course of 24 h experiments in the reduced predation treatment (**A**) but not in the ambient predation (no dilution) treatment (**B**). The grey line denotes the linear model fit.

We further tested whether predator densities were related to prey diversities within individual dilution treatments (Fig. 4A, B). The densities of HNFs, i.e. the predator group that was quantifiable by flow cytometry, were positively associated with prey diversity components in the reduced predation treatment (regression for prey richness: *R*^2^ = 0.30, *p* = 0.02; evenness: *R*^2^ = 0.32, *p* = 0.01; phylogenetic distinctiveness: *R*^2^ = 0.23, *p* = 0.03; Fig. S5). Further, during the course of the experiments, prey richness decreased less in diluted microcosms that had higher HNF densities (linear regression: *R*^2^ = 0.23, *p* = 0.03, Fig. 4A). By contrast, there was no relationship between HNF densities and richness or genetic diversity in undiluted microcosms (Fig. 4B, *p* > 0.10), and only prey evenness was positively associated with HNF densities (*R*^2^ = 0.45, *p* = 0.003).

**Fig. 5:**
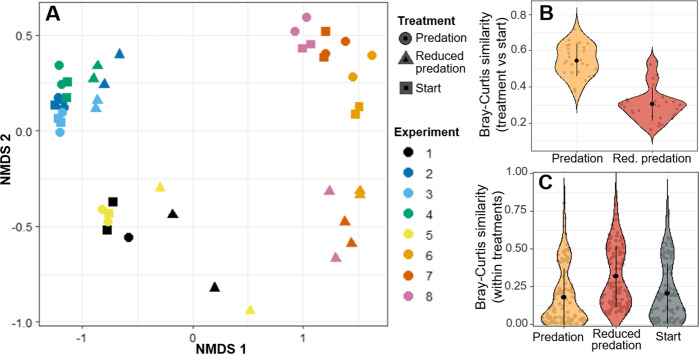
Differences in taxonomic composition of prokaryotic communities at the start and at the end of the dilution experiments. **A** Non-metric multidimensional scaling (NMDS) representation of Bray-Curtis community similarity. **B** Similarity between communities at start and in undiluted (i.e. high predation pressure) samples from the same experiment was significantly higher (*p* < 0.001) than the similarity between communities at start and in diluted (i.e. reduced grazing) samples. **C** Community similarity within treatments was significantly higher for the reduced predation treatment (*p* < 0.001), indicating reduced beta diversity and community homogenisation. Grey points in **B** and **C** represent pairwise community comparisons, black points represent means of community comparisons and the black horizontal lines are ±1 standard deviation.

Bacterial beta diversity was strongly influenced by dilution and associated reduction in predation pressure. Bacterial community composition was predominantly driven by differences in inocula, but the composition of bacterial communities also changed over time (Fig. 5A). These temporal changes were more pronounced in the diluted microcosms (Fig. 5B, C; ANOVA; *F*_(1,56)_ = 103, *p* < 0.001), leading to a homogenisation of communities illustrated as drop in beta diversity (Bray-Curtis dissimilarity) from 0.80 to 0.68 (ANOVA, *F*_(2,327)_ = 15.83, *p* < 0.001). Protozoan beta diversity, however, significantly increased from 0.76 to 0.86 in diluted microcosms (Kruskal-Wallis Test, *W* = 3140, *p* < 0.01).

**Fig. 6:**
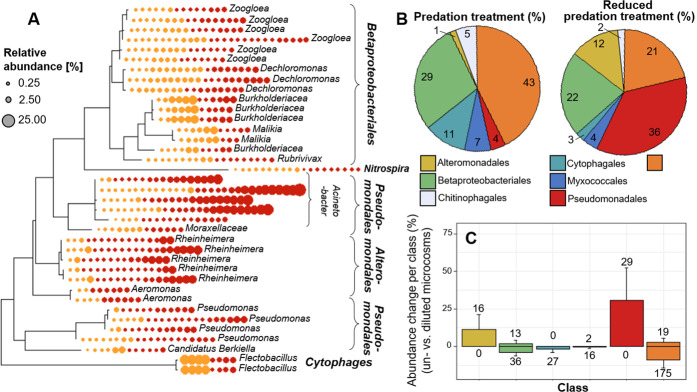
Phylogenetic tree relatedness and taxonomic identity of prokaryotic ASVs dominating reduced and ambient predation treatments. **A** A phylogenetic three showing all taxa with a mean relative abundance of >0.35% across all microcosms (*n* = 37). Circles present presence (red: reduced predation; yellow: ambient predation), size of the circle reflects relative densities. Taxonomic affiliation is expressed at the order level (bold) and at the lowest taxonomic level that could be associated to ASVs. **B** The relative contribution of different orders to the total number of reads in reduced predation and ambient predation treatments. **C** Differences in relative abundance of all taxa (summed at class level) that significantly differed between predation and reduced-predation treatments. For each order, ASVs that expressed positive and negative change were summed separately. Numbers denote the counts of ASVs with a significant difference between treatments. Bars represent standard deviation of class sums per treatment. Cytophagales did not include any ASVs that significantly differed between treatments are not displayed in **C**.

Bacterial communities in all treatments were dominated by Proteobacteria, but experimental dilution shifted dominance from Betaproteobacteriales to Pseudo- and Alteromonadales (Fig. 6A–C). Experimental dilution resulted also in an increase in HNA-LNA ratios (i.e., an increase in the relative abundance of more active cells; paired *t* test, *t* value = 3.8, *p* = 0.002; Figs. S6–8). Shifts in bacterial community composition had a substantial effect on the putative functionality of activated sludge communities. The comparison of our results with a global meta-analysis (Table 1) revealed that relative densities of many bacterial taxa associated with increased treatment efficiency, significantly declined in the low predation treatment. This included numerous taxa belonging to the Rhodocyclaceae (e.g. *Canditatus Accumulibacter*), Comamonadaceae and Nitrospiraceae families (Table 1). An exception from this observation were the families of Moraxcellaceae and Xanthomonadaceae. Whereas Xanthomonadaceae did not show much of a net change, Moraxcellaceae, a group often associated with improved aggregate formation and phosphorus removal, benefited from the experimental dilution. These findings were also corroborated by a METAGENassist analysis, showing a strong reduction in N-removal potential and a tentative reduction in C remineralisation in the reduced predation treatment (Fig. S9).

## Discussion

Despite the importance of protozoan predation for maintaining treatment efficiency in activated sludge communities [3, 4], the mechanisms governing this process are poorly understood. We demonstrated that the manipulation of protozoan predators has profound impacts on bacterial diversity and community composition with potentially far-reaching implications for ecosystem functioning. Both the decrease of prey encounter rates through dilution and the removal of top predators via filtration substantially altered bacterial prey diversity, whereas predator diversity per se had only lesser and ambiguous impacts. Moreover, reductions in predator-prey encounter rates via dilution altered bacterial community composition and triggered the decline of multiple taxa that support wastewater treatment efficiency. This suggests that protozoan predation may enhance functioning of activated sludge communities through diversity and compositional effects, which are at least partly mediated by the identity of dominant predators.

### The impact of predation pressure on prey diversity

Dilution experiments to regulate predator-prey encounter rates are common tools in plankton ecology [40, 59], but comparable, manipulative predation experiments are almost non-existent in activated sludge research. In our study, reduced encounter rates, which are well known to weaken top-down control [40], caused marked declines in richness, evenness and phylogenetic diversity of bacterial prey communities. This positive effect of predation on prey diversity is likely governed by preventing the competitive exclusion of slower growing bacteria that invest more resources in antipredator defences [Fig. 1; [32]].

Predators themselves have adapted to antipredator defences of their prey [60] causing a diversification of defence strategies such as increases in prey body size, movement speed or toxin production [61–63]. The emerging positive impact on prey diversity is often maintained by predator and prey population fluctuations, density-dependent predation and diversity-enhancing ‘kill the winner’ dynamics [i.e. reducing the dominance of successful competitors; [39]]. Specialist predators can support such ‘kill the winner‘ dynamics because of their high susceptibility to food limitation. Therefore, changes in prey population can cause even at the very high food densities found in activated sludge reactors that predators enter the non-linear part of their functional response curves, enforcing density-dependent prey control [64–66]. Generalist predators, on the other hand, often preferentially feed on the most common prey types, again triggering ‘kill the winner‘ dynamics [67, 68]. Hence, a positive response of prey diversity to predation is not only based on the resulting co-existence of *K-* and *r*-strategists, but also emerges from density-dependent predation and from the co-existence of multiple *K*-strategists with alternative predator-defence mechanisms.

However, an increase in predation pressure does not necessarily result in a linear, positive impact on prey diversity [27, 28]. We found the effect of predation on prey diversity to vary along a gradient of predation intensity. Whereas HNF densities were positively associated with bacterial diversity in the reduced predation treatment, there was no clear association in undiluted microcosms with high predation pressure. Even though HNFs represent only one group of predators in activated sludge communities, these findings support previous hypotheses of a hump-shaped relationship between prey diversity and predation pressure [28, 32]. The predation intensity that results in maximal prey diversity (i.e. the peak of the hump) has been suggested to increase with ecosystem productivity [Fig. 1; [27]]. In highly productive activated sludge communities, this may result in an overall positive impact of protozoan biomass on prey diversity. However, protozoans can account for very high proportion of community biomass, reaching up to 20% of total activated sludge mass [69]. Such elevated predator biomass may eventually exceed limits of beneficial top-down control and trigger negative responses in prey diversity.

### Diversity effects on ecosystem functioning

Positive impacts of diversity on functioning are well supported across ecosystem types and taxonomic groups [21, 70] and hence high bacterial diversity can be expected to also increase wastewater treatment efficiency [e.g. enhanced nutrient-uptake, reduced biological oxygen demand in outflow; [7, 71]]. Research about diversity and ecosystem functioning traditionally relied on species richness as biodiversity indicator [72]. However, it has been argued that phylogenetic diversity is a better predictor of functionality as it better reflects niche complementarity, a key mechanism linking biodiversity to ecosystem functioning [73]. Here, we used phylogenetic distinctiveness as a measure of phylogenetic diversity because of its mathematical independence from taxa richness [54]. Nevertheless, we showed that losses of phylogenetic diversity resulting from reduced predation pressure were mitigated by high taxa richness (Fig. 3D). These findings agree with the insurance hypothesis, postulating that high taxa richness mitigates the erosion of functionality in stressed ecosystems [74]. Therefore, the insurance hypothesis may be an important mechanism enhancing treatment efficiency in activated sludge reactors with high bacterial diversity.

Beta diversity represents another biodiversity component that can improve ecosystem functioning, particularly at larger spatial and temporal scales [75, 76]. We showed that beta diversity was positively related to high predation pressure (Fig. 5). By contrast, conceptual frameworks [32] and experiments with fish communities [29, 77] suggested a negative impact of predation on beta diversity. In this context, predation is suggested to reduce stochasticity and increase the relative importance of deterministic community assembly processes [29]. The contrasting results in our study may result from our focus on complex and highly variable predator assemblages compared to the previous work that investigated the impacts of a single top predator [29, 77]. Protozoan predators show a high functional diversity in their feeding modes [63, 78] and therefore impose different selection pressures on their prey [e.g. ambush vs. filter feeding predators; [60]]. Hence, predation in our study may still have enhanced the importance of deterministic assembly processes [29]. However, diverging selection pressures across our experiments would “push” prey communities in different directions, explaining the observed increase in beta diversity in our study.

### The effects of community composition on ecosystem functioning

Dilution of microcosms resulted in strong changes in the identity of dominant bacterial ASVs in our experiments. These changes can in principle emerge from reductions in predator-prey encounter rates and predation pressure or from an increased resource supply in diluted communities. Dilution experiments are designed to maintain an equal initial resource availability across treatments [40], which together with the high resource concentration in the growth media counteracts resource limitation. Moreover, if nutrient limitation was an important driver of community changes, it should have had a stronger impact in undiluted microcosms. Yet, these differences were small compared to temporal changes in community composition in diluted microcosms and therefore differences in resource availability likely played a subordinate role in driving community shits.

At higher taxonomic levels, ASVs belonging to the same taxon exhibited partly contrasting responses to reduction in predation pressure (Fig. 6). Diverse responses can generally be expected because of the high functional diversity within higher taxonomic groups (e.g. Betaproteobacteriales) and predation-mediated changes in the outcome of competition among closely related prey species. Despite these sometime bi-directional changes, our assessment of putative functionality in sludge communities, a topic that currently gains rapidly in attention [79], indicated decreases of treatment efficiency at lower levels of predation pressure. Relative densities of many taxa that are associated with high wastewater treatment efficiency, such as Comamonadaceae, *Nitrospira* and *Candidatus Accumulibacter* [6, 7] increased in treatment with high predation pressure (Table 1). Compositional changes resulted in a tendency of a decreasing potential for carbon degradation and phosphorus uptake and a strong reduction in nitrogen removal at low predator-prey encounter rates (Table 1, Fig. S9). Although these findings are restricted to putative functionality, they highlight the large potential impacts that changes in predation may have on wastewater processing in activated sludge communities.

### Outlook

The overarching goal of many recent studies and research applications is to maximise the positive impacts of bacterial communities on wastewater treatment efficiency [2, 5, 7]. Our findings demonstrate the critical role of protozoan predation in governing diversity and composition of activated sludge communities and suggest their indirect consequences for treatment efficiency. We call for more community-level experiments that directly manipulate mechanisms linking predator and prey density, identity, and multiple aspects of diversity with specific functions of activated sludge ecosystems. Such mechanistic research represents a crucial step forward in advancing general ecological theory as well as improving the capacity of biological treatments in activated sludge reactors.

## Supplementary information

Supplementary information

Annex - associated R code
