# Supplementary material for: Predation increases multiple components of microbial diversity in activated sludge communities
Source: ISME J. 2021 Dec 1;16(4):1086–94. doi: 10.1038/s41396-021-01145-z (PMC8941047; doi:10.1038/s41396-021-01145-z)
Supplement: Supplementary file 1 — Supplementary information [file 41396_2021_1145_MOESM1_ESM.docx]

**Supplementary Information**

**Predation increases multiple components of microbial diversity in activated sludge communities**

Alfred Burian, Daisy Pinn, Ignacio Peralta-Maraver, Michael Sweet, Quentin Mauvisseau, Ozge Eyice, Mark Bulling, Till Röthig, Pavel Kratina

**Section S1: Flow cytometry method description**

HNF densities and HNA-LNA bacteria ratios were analysed on a BD Accuri™ C6 automatic flow cytometer (BD Biosciences, USA). First, validation beads were used for flow cytometer calibration establishing thresholds for side scatter and florescence intensities. Sample preparation involved pre-sieving through a 40µm nylon cell strainer (Corning Inc., US) and staining with SYBR Green I (10%) in dimethyl sulfoxide. Time periods between defrosting and measurements were restricted to <10 min to ensure method reliability. Both, HNA and LNA cells were discriminated based on side scatter as well as green and red fluorescence (Fig. S9), following [1]. Flow speed was set to 10μL min^-1^ for 120 seconds, and dilutions were chosen to ensure that the mean number of events stayed below 1,000 min^-1^. In order to quantify HNF densities, 125μL samples were diluted in 875µL of Milli-Q water and 10μL of SYBR Green dimethyl sulfoxide. All samples were processed on the flow cytometer for 5 minutes at 100µL min^-1^. Green and red fluorescence were used to distinguish HNFs from chlorophyll-containing protists.

The chemostat set-up is in certain way mimicking the design of activated sludge reactors, but there are also substantial differences, which limit direct comparisons. A key difference is that activated sludge reactors are fed with a mixture of inflow water and recycled activated sludge (volume ratios can largely differ among reactors but roughly vary around ratios of 1:1). This allows substantially the amount of inflow that can be handled by activated sludge reactors without risking that bacterial communities are flushed out. On the other hand, sludge retention times can be quite high in activated sludge reactors (4-8 days if P-update is the main target, increases to ~2 weeks if N should also be removed effectively).


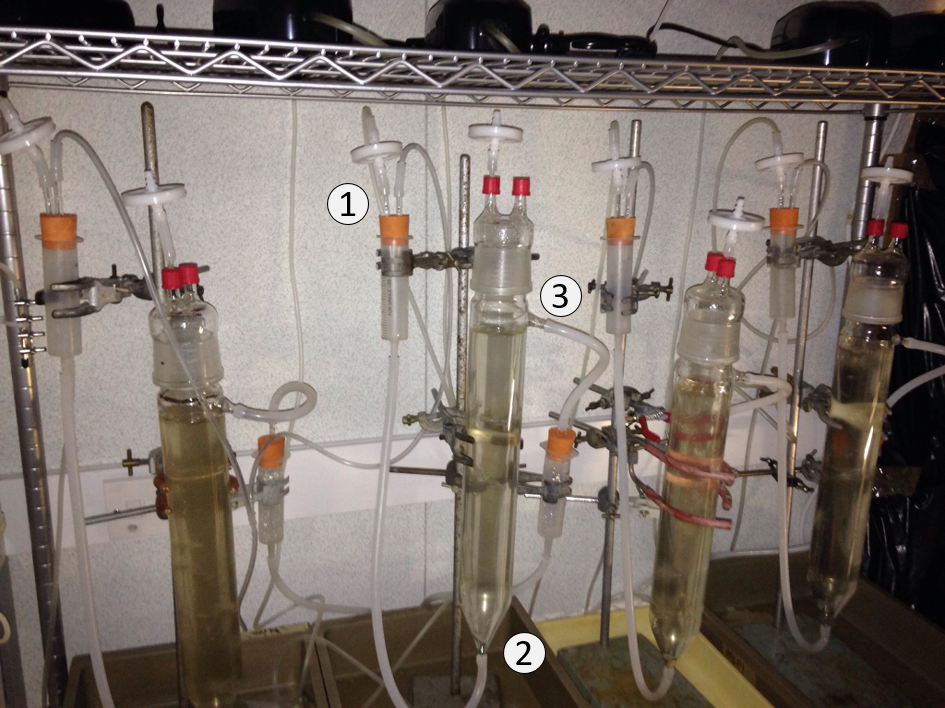


**Fig. S1:** Set-up of chemostat cultures. (**1**) The media inflow and the aeration pipe are joint together. (**2**) Inflow in chemostat is integrated at the bottom of chemostats to avoid sedimentation of particles. (**3**) Outflow of the cultures in a microbial trap avoids contamination from the outflow bottle. All air inflow and outflow was filtered through 0.2 μm air filters to prevent contamination of cultures. Chemostats were 1L in volume and operated at different dilution rates. At the start of the conditioning phase, chemostat were inoculated with 100mL of activated sludge and 500 mL of media (the additional 400mL dropped in continuously once the medium flow was switched on). Community biomass in chemostats did not reach the very high values frequently observed in activated sludge reactors as there are systematic difference in functional design (e.g. no change between aerobic and anaerobic cycle in chemostats). However, our chemostats set-up had the advantage to be operated on sterilised media and hence potential confounding influences of immigrating microbial communities could be excluded in our experiments. Cultures were kept in in darkness at 20˚C.


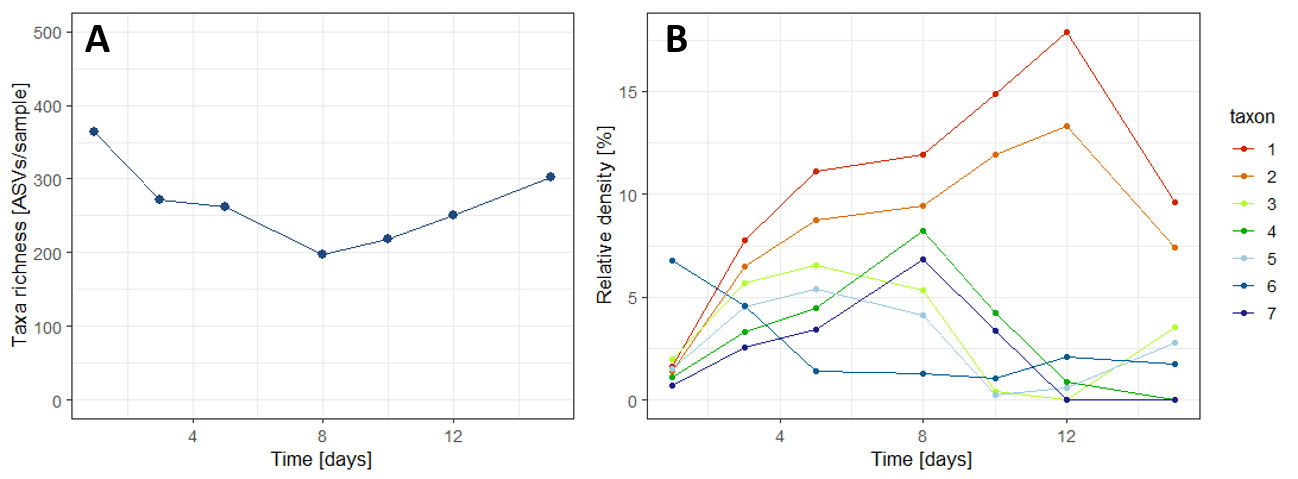
**Fig. S2:** Dynamics of the 7 most abundant ASVs contributing to prey communities (**A**) and total richness (**B**) in chemostat during the incubation period. Total community composition showed strong fluctuations but a marked change from starting conditions. Overall richness was largely maintained over the entire incubation period. Changes in total richness after day 8 are likely an artefact of next-generation sequencing analysis and the failure of detecting highly rare species. Therefore, increases in evenness can result in slight increases in total richness without external contamination.


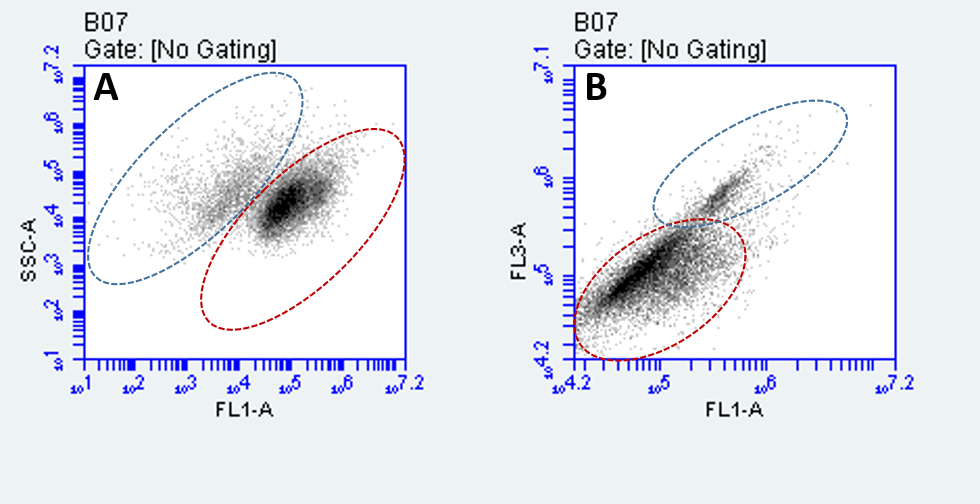


**Fig. S3:** Visualisation of flow cytometer data. The combined measures of green fluorescence (FL1-A) and side scatter (SSC-A) in panel (**A**) and green and red fluorescence (FL3-A) in panel (**B**) allowed to differentiate between HNF (blue circle) and LNF (red circle).


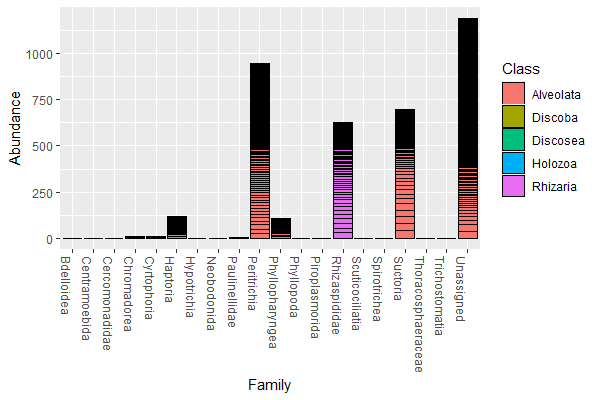


**Fig. S4:** Abundance expressed as the mean number of sequence reads per sample of different families of protozoan predators detected in our study. Stacked bars for each family show the relative abundance of individual ASVs. The colour scheme indicates their class affiliation. Taxonomy is based on SILVA data-base 2020 release.


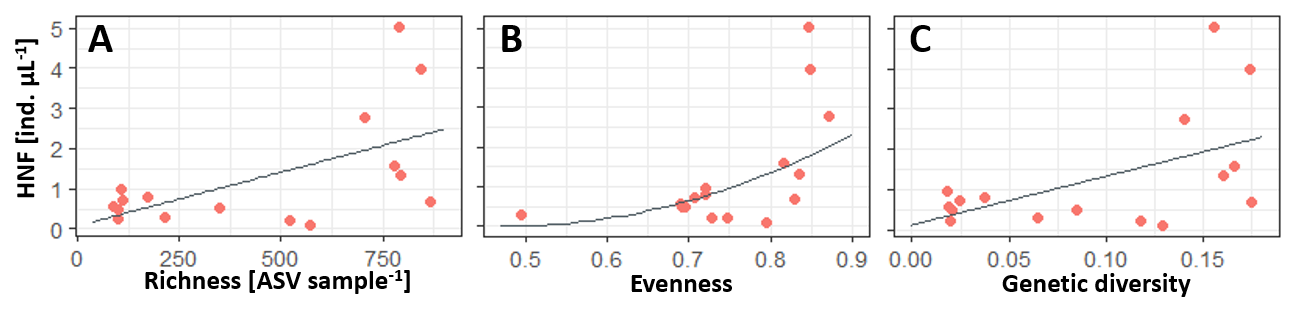


**Fig. S5:** Relationships between density of heterotrophic nanoflagellates (HNFs) at the end of the experiment and several diversity indices in diluted samples (reduced grazing) (**A**: Richness **B**: Evenness **C**: Genetic diversity).


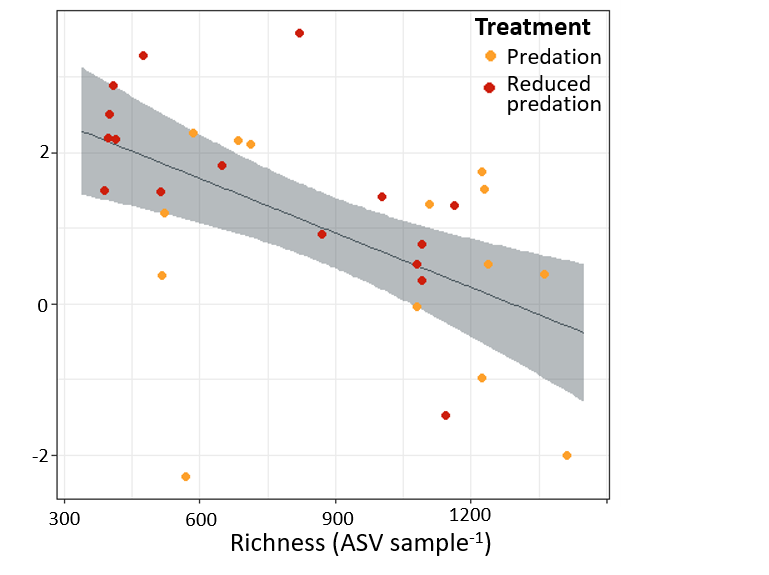


**Fig. S6:** Relationship between ln-transformed HNA:LNA ratio of bacterial community and bacterial ASV richness at the end of the study. The grey line denotes the model fit, the light-grey area illustrates the 95% confidence interval of the slope.

**
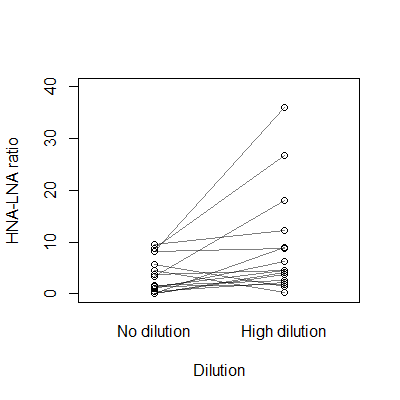
Fig. S7:** Differences in HNA-LNA ratios between diluted (reduced grazing) and non-diluted grazing. Grey lines link samples from the same experiment.


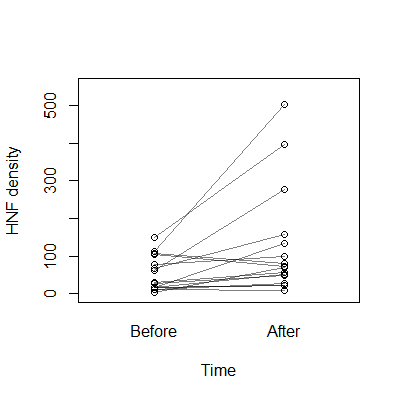


**Fig. S8:** Changes in HNF densities during the course of the experiments in non-diluted samples. Grey lines link samples from the same experiment.


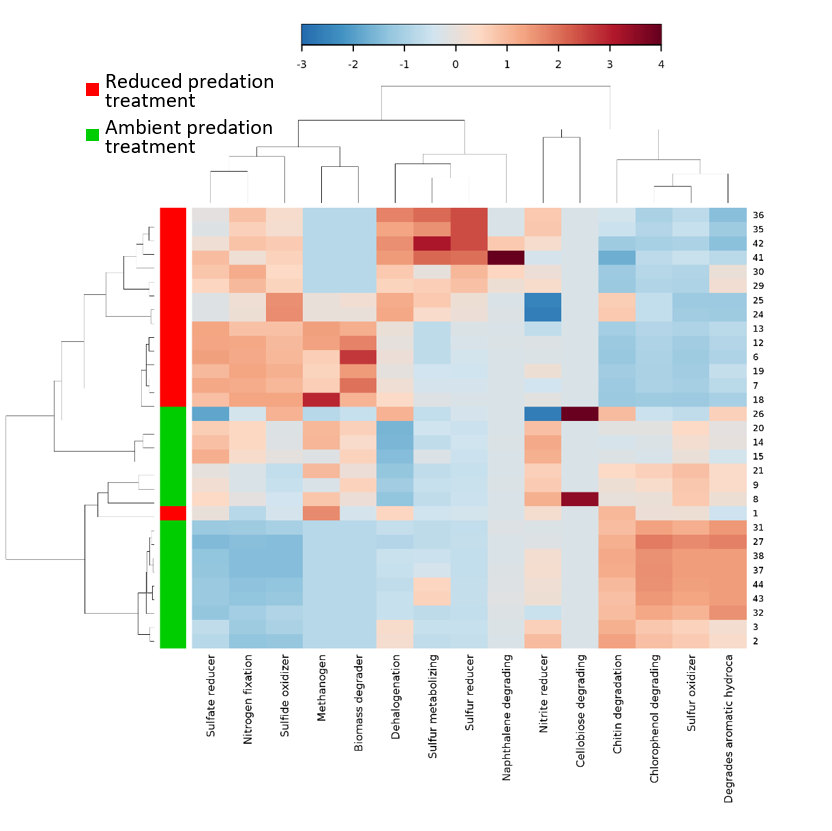


**Fig. S9:** Phylogeny-based predictions of functional differences between bacterial communities in diluted and undiluted microcosms*.* Higher levels of putative functionality are depicted in red and lower levels in blue on a z-standardised scale. Predictions were based on phylogenetic inference of the bacterial communities using METAGENassist [2]. To perform the ‘taxonomic-to-phenotypic mapping’ we employed ASV abundance table and metadata based on the Qiime2 analysis. In METAGENassist ASVs from the 31 samples were taxonomically assigned and mapped, condensed into 769 functional taxa, and filtered based on interquartile range [3]. The remaining 577 functional taxa were normalized across samples by sum and over taxa by range scaling. We analysed the dataset for ‘metabolism by phenotype’ using the Pearson distance measure, clustered using the average algorithm, and selected the 15 most differentially abundant metabolic processes by random forest.

**Table S1:** Summary statistics of bacterial and protozoan diversity metrics according to the experimental treatment manipulations (P = ambient predation treatment, RP = reduced predation treatment, S = start samples) and the implementation of pre-filtering in the conditioning phase. Values represent means and one standard deviation (in brackets). Letters and colours of cells indicate significant differences among groups of categorical response variables.

| Sample groups | Bacterial taxa richness | | Bacterial taxa evenness | | Bacterial genetic distinctiveness | | Protozoan taxa richness | | Protozoan taxa evenness | | Protozoan genetic distinctiveness | |
| --- | --- | --- | --- | --- | --- | --- | --- | --- | --- | --- | --- | --- |
| P | 696 | (±354)^a^ | 0.84 | (±0.07)^a^ | 12.81 | (±1.33)^a^ | 72.3 | (±16.9)^a^ | 0.71 | (±0.07)^a^ | 4.50 | (±0.90)^a^ |
| RP | 444 | (±316)^b^ | 0.75 | (±0.09)^b^ | 11.24 | (±2.93)^b^ | 42.2 | (±10.3)^b^ | 0.72 | (±0.13)^a^ | 4.22 | (±1.05)^a^ |
| S | 727 | (±386)^a^ | 0.85 | (±0.06)^a^ | 12.81 | (±1.45)^a^ | 90.5 | (±26.1)^c^ | 0.72 | (±0.07)^a^ | 4.66 | (±0.99)^a^ |
| Pre-filtered | 239 | (±112)^x^ | 0.76 | (±0.06)^x^ | 13.66 | (±0.46)^x^ | 73.7 | (±28.4)^x^ | 0.72 | (±0.06)^x^ | 3.50 | (±0.42)^x^ |
| Unfiltered | 863 | (±246)^y^ | 0.85 | (±0.08)^y^ | 10.09 | (±1.94)^y^ | 68.8 | (±27.4)^x^ | 0.70 | (±0.12)^x^ | 5.24 | (±0.42)^y^ |
| Total | 619 | (±368) | 0.81 | (±0.09) | 12.26 | (±2.16) | 71.5 | (±27.7) | 0.72 | (±0.09) | 4.49 | (±0.97) |

**Table S2:** Summary statistics of the regression analysis examining the drivers of bacterial and protozoan diversity. Treatment abbreviations represent reduced grazing (RG), normal grazing (G) and start of the experiment (S). Different dimensions of protozoan diversity were used as predictors of bacterial diversity, although we want to acknowledge that influences are best conceptualised as bidirectional. All models have been fitted as a general linear regression except of the model for bacterial phylogenetic distinctiveness, which was fitted as a generalised least square regression setting varIdent as (form=~1|Treatment*Filtration) in the nlme package in R, which led to the model with the lowest AIC value.

| **Dependent variable** | **Model** | **Description** | ***r*^2^** |
| --- | --- | --- | --- |
| Bacterial richness | y = -599x_1_ + x_2_ + 918 | x_1_ = Filtration (p<0.001), x_2_ = Treatment (*p*<0.001 for G-RG and RG-S, n.s. for G-S; factor values: G: 0, RG: -311, S: 56) | 0.82 |
| Bacterial evenness | y = -0.07x_1_ - 0.21x_2_ + x_3_ +1.00 | x_1_ = Filtration (*p*<0.01), x_2_ = protozoan evenness (*p*=0.088), x_3_ = Treatment (*p*<0.001 for G-RG and RG-S, n.s. for G-S; factor values: G: 0, RG: -0.11, S: 0.03) | 0.56 |
| Bacterial phyl. distinctiveness | y = 0.0089x_1_ - 6.45x_2_ -0.598x_3_ - 3.54x_4_ + x_5_ + 20.9 | x_1_ = protozoan richness (*p*=0.013), x_2_ = protozoan evenness (*p*<0.001), x_3_ = protozoan distinct. (*p*=0.028), x_4_ = Filtration (*p*<0.001), x_5_ = Treatment (*p*<0.001 for G-RG and RG-S, n.s. for G-S; factor values: G: 0, RG: -3.08, S: 0.13) | 0.55 |
| Protozoan richness | y = x + 72.3 | x = Treatment (*p*<0.001 for RG-S, *p*=0.0013 for G-RG, *p*=0.025 for G-S; factor values: G: 0, RG: -30.1, S: 18.1) | 0.48 |
| Protozoan evenness | y = 1 |  | n.s. |
| Protozoan phyl. distinctiveness | y = -1.74x + 5.24 | x = Filtration (*p*<0.001) | 0.81 |

**References**

1. Gasol JM, Morán XAG (2015). Flow cytometric determination of microbial abundances and its use to obtain indices of community structure and relative activity. *Hydrocarbon and Lipid Microbiology Protocols*. Springer. pp 159-187.

2. Arndt D, Xia J, Liu Y, Zhou Y, Guo AC, Cruz JA *et al* (2012). METAGENassist: a comprehensive web server for comparative metagenomics. *Nucleic Acids Res* **40:** W88-W95.

3. Hackstadt AJ, Hess AM (2009). Filtering for increased power for microarray data analysis. *BMC bioinformatics* **10:** 1-12.
