# Supplementary material for: Predation increases multiple components of microbial diversity in activated sludge communities
Source: ISME J. 2021 Dec 1;16(4):1086–94. doi: 10.1038/s41396-021-01145-z (PMC8941047; doi:10.1038/s41396-021-01145-z)
Supplement: Supplementary file 2 — Annex - associated R code [file 41396_2021_1145_MOESM2_ESM.pdf]

```
library("ggplot2"); library(GUniFrac); library(plyr); library(stringi); library(ecodist); library(PhyloMeasures); library
(nlme)
library(car); library(xlsx); library(vegan); library(geiger); library(jcolors); library(sinaplot);library("ggforce")
#source("https://bioconductor.org/biocLite.R") # Installation lines
#biocLite("biomformat")
library(biomformat)
#source('http://bioconductor.org/biocLite.R')
#biocLite('phyloseq')
library(phyloseq)

# reading in files
otu<-read.table(file = 'out_table.txt', sep = '\t', header = TRUE)
taxonomy<-read.table(file = 'taxonomy.tsv', sep = '\t', header = TRUE)
tree<- read.tree("tree.nwk")
meta<- read.csv('meta.csv',header=T, dec=".",sep=",",na.strings="NA")# has the same first colum as the first row of
otu

#first - align taxonomy and otu table
colnames(otu)<- c("OTU.ID", as.character(meta$OTU.ID))
taxonomy<-taxonomy[match(otu$OTU.ID, taxonomy$Feature.ID), ] # but anyhow, it was the same order

# split taxonomy
taxonomy.split<-strsplit(as.character(taxonomy$Taxon),';') #split up into a list
max.length <- max(sapply(taxonomy.split, length))
a<-lapply(taxonomy.split, function(v) { c(v, rep(NA, max.length-length(v)))}) # fill up with NAs
taxonomy.split<-do.call(rbind, a)
otu.tax<-cbind(otu,taxonomy.split)
names(otu.tax)<-c(colnames(otu),"Kingdom","Phylum","Class","Order","Family","Genus","Species")

# Remove NAs and turn them into unassigned
s<-data.frame(lapply(otu.tax[,93:99], as.character), stringsAsFactors=FALSE)
s[,1]<-as.character(s[,1])
for (i in 1:ncol(s)) {s[,i][is.na(s[,i])] <- "xxxxUnassigned"}
# Get rid of the letters beofre taxa names
for (i in 1:nrow(s)){if(s[i,1]!="Unassigned"){s[i,1]<-stri_sub(s[i,1],4)}}
for (i in 1:nrow(s)){for (k in 2:ncol(s)) {s[i,k]<-stri_sub(s[i,k],5)}}
for (i in 1:ncol(s)) {s[,i][s[,i]==""] <- "Unassigned"}

# remove chloroplasts + weird taxa that are not assigned
a<-cbind(otu,s)
a<-subset(a,a$Class!="Chloroplast" & a$Kingdom!="Unassigned") # only few chloroplast, but they are removed
otu<-a[,2:92]; rownames(otu)<-as.character(a[,1]);  colnames(otu)<- c(as.character(meta$OTU.ID))
taxonomy<-rbind(a[,93:99]); rownames(otu)<-as.character(a[,1])

# select which data you want to work with - time: 99 represents results of dilution experiment (=Daisy)
water.otu<-otu[,which(meta$experiment=="water")]
otu<-otu[,which(meta$time==99)]; meta<-meta[which(meta$time==99),]

# get rid of samples with too low reads
x<-c()
for (k in 1:ncol(otu)){x<-c(x,sum(otu[,k]))}
otu<-otu[,-which(x<15000)]; meta<-meta[-which(x<15000),]

#calculate relative densities in %
sums<-c()
```

```
for(i in 1:ncol(otu)){x<-sum(otu[,i])
sums<-c(sums,x)}

column<-c(); otu.relative<-c()
for (i in 1:ncol(otu)){
  for (k in 1:nrow(otu)){
    x<-otu[k,i]/sums[i]
    column<-c(column,x)}
  otu.relative<-cbind(otu.relative,column)
  column<-c()}
otu.relative<-as.data.frame(otu.relative)*100; colnames(otu.relative)<- c(as.character(meta$OTU.ID))


# account for blank control - they way this is done is that I set a threshold for abundant taxa, which might also
# cause contamination in real samples and then eliminating these taxa from real samples if they are present at low
# abundance (they would be present at low abundance because contamination is low compared to sample DNA, at le
ast
# it should be!)
x<-rowSums(water.otu)/sum(colSums(water.otu))*100; a<-c(0)
for (k in 1:ncol(otu)){
for (i in as.numeric(which(x>0.05))) # treshold: potential candidates for contamination in real samples: >0.05% rel.
den.
{if(otu.relative[i,k]<0.1 & otu.relative[i,k]!=0){otu[i,k]<-0; a<-a+1}}} # a is a count of how many species are throw
n out

#calculate relative densities in % again after cleaning out stuff
sums<-c()
for(i in 1:ncol(otu)){x<-sum(otu[,i])
sums<-c(sums,x)}

column<-c(); otu.relative<-c()
for (i in 1:ncol(otu)){
  for (k in 1:nrow(otu)){
    x<-otu[k,i]/sums[i]
    column<-c(column,x)}
  otu.relative<-cbind(otu.relative,column)
  column<-c()}
otu.relative<-as.data.frame(otu.relative)*100; colnames(otu.relative)<- c(as.character(meta$OTU.ID))


##########################################################################################
#####################
# for some calculations is best to use rarefied data, for some it is best to use relative data.
# consequently, otu is processed in both ways

x<-c()
for (k in 1:ncol(otu)){x<-c(x,sum(otu[,k]))}
a<-min(x) # minimum number of species/Sample

otu.rarifyed<- otu[,1:ncol(otu)]
for (k in 1:ncol(otu)){
  for (i in 1:nrow(otu)){otu.rarifyed[i,k]<-otu[i,k]*(a/x[k])}}

otu.rarifyed.rd<-trunc(otu.rarifyed)
commas<-otu.rarifyed-otu.rarifyed.rd
```

```
upround<-c()
for (k in 1:ncol(otu)){upround<-c(upround,sum(commas[,k]))} #determines how many species must be rounded up
per sample

rank<-c()
for (k in 1:ncol(otu)){i<-order(-commas[,k]) # determines the rows with the highest numbers after the comma
rank<-cbind(rank,i)}

for (k in 1:ncol(otu)){
  for (i in 1:upround[k]){otu.rarifyed[rank[i,k],k]<-otu.rarifyed.rd[rank[i,k],k]+1}} # round up what needs to be roun
d up
otu.rarifyed<-trunc(otu.rarifyed)

rm(commas,s, otu.tax,otu.rarifyed.rd,rank,taxonomy.split)


#calculate relative densities in % for rarefied samples
sums<-c()
for(i in 1:ncol(otu)){x<-sum(otu.rarifyed[,i])
sums<-c(sums,x)}

####################################################################################################
########
######calcualte species richness#############
s<-0
richness<-c()
for (k in 1:ncol(otu.rarifyed)){
  for(i in 1:nrow(otu.rarifyed)){
    if(otu.rarifyed[i,k]==0){x<-0} else {x<-1}
    s<-s+x}
  richness<-c(richness,s)
  s<-0}

######calcualte average reads #############
av_no_reads<-c()
for (k in 1:ncol(otu)){x<-sum(otu[,k])/richness[k]
av_no_reads<-c(av_no_reads,x)}

######calcualte simpsons diversity index#############
s<-0
simpson<-c()
for (k in 1:ncol(otu.rarifyed)){
  for(i in 1:nrow(otu.rarifyed)){
    if(otu.rarifyed[i,k]<1.1){x<-0} else {x<-otu.rarifyed[i,k]/sum(otu.rarifyed[,k])*(otu.rarifyed[i,k]-1)/
      (sum(otu.rarifyed[,k])-1)}
    s<-s+x}
  simpson<-c(simpson,1-s)
  s<-0}

######calcualte Shanon diversity #############
s<-0
shannon<-c()
for (k in 1:ncol(otu.rarifyed)){
  for(i in 1:nrow(otu.rarifyed)){
```

```
    if(otu.rarifyed[i,k]==0){x<-0} else {x<-otu.rarifyed[i,k]/sum(otu.rarifyed[,k])*
      log(otu.rarifyed[i,k]/sum(otu.rarifyed[,k]))}
    s<-s+x}
  s<-s*(-1)
  shannon<-c(shannon,s)
  s<-0}

######unweigthed faith - genetic diversity #############

s<-0
faith<-c()
for (k in 1:ncol(otu.rarifyed)){
  # drop.tip drops a certain number of elements from a tree. In order to keep a certain number of elements
  # to whole match thing is necessary
  pruned.tree<-drop.tip(tree, tree$tip.label[-match(rownames(otu.rarifyed)[which(otu.rarifyed[,k] > 0)], tree$tip.label
)])
  total.tree<-drop.tip(tree, tree$tip.label[-match(rownames(otu.rarifyed)[which(rowSums(otu.rarifyed)>0)],tree$tip.la
bel)])
  s<-sum(pruned.tree$edge.length)/ sum(total.tree$edge.length)
  faith<-c(faith,s)}

######## genetic distinctiveness #############
faith.c<-c(); s<-0
for (k in 1:ncol(otu.rarifyed)){
  pruned.tree<-drop.tip(tree, tree$tip.label[-match(rownames(otu.rarifyed)[ which(otu.rarifyed[,k] > 0)],
                                                    tree$tip.label)])
  total.tree<-drop.tip(tree, tree$tip.label[-match(rownames(otu.rarifyed)[ which(rowSums(otu.rarifyed) > 0)],
                                                   tree$tip.label)])
  s<-pd.moments(pruned.tree, min(richness))
  faith.c<-c(faith.c,s[1,1])}

######calcualte evenness #############
Pielous.evenness<-shannon/log(richness)

##############################################################################################
##################
##############################################################################################
##################
##############################################################################################
##################
##############################################################################################
##################
###### 1) Richness

#overview plot - not standardised by start
plot_dat<-cbind(meta, richness, Pielous.evenness, faith, faith.c)
plot_dat<-subset(plot_dat, plot_dat$experiment!="blank"& plot_dat$experiment!="start")

colour_code3<- c(rgb(253,160,43,maxColorValue=255), rgb(205,31,13,maxColorValue=255),rgb(72,85,92,maxCol
orValue=255))

#point plot
ggplot(data = plot_dat, aes(x=experiment, y = faith.c, group = treatment, colour = treatment, shape =treatment,
                            fill =treatment))+ labs(y = "Phylogenetic distinctiveness", x = "Experiment")+
  theme_bw(base_size = 13)+ geom_point(size=1.9)+ scale_shape_manual(values=c(25,22,24))+
```

```
  scale_color_manual(values=colour_code3)+scale_fill_manual(values=colour_code3)

#stat test
library(nlme);library(MuMIn)
plot_dat$treatment<-relevel(plot_dat$treatment,"C2")
mod1<-lme(Pielous.evenness~ treatment, random = ~ 1 | experiment, data=plot_dat)
anova(mod1); r.squaredGLMM(mod1)
summary(mod1)
str(plot_dat)

# get the C1 and C1 standardized by start to account for systematic differences between experiments
# first get mean of start; one could also say does not work with the mean - but after thinking about it I think that
#standardising by the mean is more robust and not artificially blowing up the results...

a<-subset(plot_dat, plot_dat$treatment!="start") # get data that needs standardisation
for (i in 1:nrow(a)){x<-a$experiment[i]
a$richness[i]<-a$richness[i]-mean(plot_dat$richness[which(plot_dat$treatment=="start" & plot_dat$experiment==x
)])
a$Pielous.evenness[i]<-a$Pielous.evenness[i]-mean(plot_dat$Pielous.evenness[
  which(plot_dat$treatment=="start" & plot_dat$experiment==x)])
a$faith[i]<-a$faith[i]-mean(plot_dat$faith[which(plot_dat$treatment=="start" & plot_dat$experiment==x)])}

## violin plots - I don't think that the display is really convincing, the point plots seem in this case better...
ggplot(data = a, aes(x=treatment, y = richness, group = treatment, colour = experiment, fill =treatment))+
  theme_bw(base_size = 15) +  geom_sina(colour = "light gray")+  geom_violin(trim = FALSE, colour = "black",  al
pha=0.6) +
  stat_summary(    fun.data = "mean_sdl",  fun.args = list(mult = 1),
                 geom = "pointrange", color = "black"  )+  labs(y = "Richness [ASVs]", x = "")

### compute relationship between phy. destinctiveness decrease and biodiversity in the treatments.
# better to do this for each experiment instead of for each replicate

destinct<-aggregate(list(plot_dat$faith.c, plot_dat$richness, plot_dat$Pielous.evenness) ,by=list(plot_dat$experimen
t, plot_dat$treatment),function(v){mean(v)})
colnames(destinct)<-c("experiment","treatment","faith.c", "richness","evenness")
destinct$experiment<-as.numeric(as.character(destinct$experiment))
mean((destinct$richness[which(destinct$treatment=="C1")]-destinct$richness[which(destinct$treatment=="C2")])/
  destinct$richness[which(destinct$treatment=="C1")])
mean((destinct$evenness[which(destinct$treatment=="C1")]-destinct$evenness[which(destinct$treatment=="C2")])/

      destinct$evenness[which(destinct$treatment=="C1")])

destinct.difference<-c();b<-c(); x<-c(); y<-c()
for (i in 1:8){a<-destinct$faith.c[which(destinct$experiment==i& destinct$treatment=="C2")]-
                                    destinct$faith.c[which(destinct$experiment==i& destinct$treatment=="start")]
            destinct.difference<-c(destinct.difference,a)
            b<-c(b,destinct$richness[which(destinct$experiment==i& destinct$treatment=="start")])}
p<-data.frame(destinctiveness = destinct.difference, Richness = b)

mod1<-lm(destinctiveness~Richness, data=p)
summary(mod1)

pred <- data.frame(predict(mod1, p,interval = "confidence"), Richness=p$Richness)
colnames(pred)[1]<-c("destinctiveness")
```

```
ggplot(data = pred, aes(x=Richness, y = destinctiveness))+
  geom_ribbon(aes(ymin= lwr, ymax = upr), alpha=0.4,show.legend = F,fill=colour_code3[2])+
  geom_line(color=colour_code3[3],data = pred, aes(x=Richness, y=destinctiveness),size =0.7)+
  theme_bw(base_size = 15) +  geom_point(data = p, aes(x=Richness, y = destinctiveness), colour = "black", size =1.
5)

###############################################################################################
##################
###############################################################################################
##################
###### 2) Abundant strains
#plot B - distribution of abundance

a<-otu.relative[,which(meta$treatment!="start")]
num<-100   # get 100 most abundant taxa
k<-otu.relative[order(-rowSums(a))[1:num],]
i<-rowMeans(k[,which(meta$treatment=="C1")]) - rowMeans(k[,which(meta$treatment=="C2")]) # calculate differ
ence in
sum(i[which(i<0)]);sum(i[which(i>0)])

# calculate sd for the differences
x<-cbind(rank(-i), i, taxonomy$Class[order(-rowSums(a)[1:num])],s) # merge into dataframe
for(i in 1:nrow(x)){if(x[i,3]!="Alphaproteobacteria"&x[i,3]!="Betaproteobacteria"&x[i,3]!="Gammaproteobacteria"
&
                    x[i,3]!="Deltaproteobacteria"&x[i,3]!="Cytophagia"){x[i,3]<-"others"}}
colnames(x)<-c("rank","Rel_ab", "Taxonomy","sd");x<-as.data.frame(x)
x$sd<-as.numeric(as.character(x$sd));x$Rel_ab<-as.numeric(as.character(x$Rel_ab))
x$rank<-as.numeric(as.character(x$rank))


colour_code<- c(rgb(155,187,88,maxColorValue=255), rgb(31,72,123,maxColorValue=255),rgb(149,179,215,maxC
olorValue=255),
             rgb(192,80,70,maxColorValue=255))
colour_code5<- c( rgb(119,94,240,maxColorValue=255),rgb(100,143,255,maxColorValue=255),rgb(221,38,128,ma
xColorValue=255),
             rgb(254,97,0,maxColorValue=255), rgb(255,176,1,maxColorValue=255))
colour_code9<- c( rgb(51,34,136,maxColorValue=255),rgb(18,119,51,maxColorValue=255),rgb(68,171,154,maxCo
lorValue=255),
              rgb(136,205,238,maxColorValue=255), rgb(220,204,119,maxColorValue=255), rgb(204,101,118,maxCo
lorValue=255),
              rgb(170,68,152,maxColorValue=255), rgb(136,34,84,maxColorValue=255))
colour_code9b<- c( rgb(0,0,0,maxColorValue=255),rgb(229,159,1,maxColorValue=255),rgb(86,180,232,maxColor
Value=255),
              rgb(0,159,115,maxColorValue=255), rgb(240,228,66,maxColorValue=255), rgb(0,114,177,maxColorVal
ue=255),
              rgb(213,94,0,maxColorValue=255), rgb(204,121,167,maxColorValue=255))


ggplot(data = x, aes(x=rank,  y= Rel_ab, group = Taxonomy, colour = Taxonomy,
                     fill =Taxonomy))+ labs(y = "Relative abundance change [%]", x = "")+
  theme_bw(base_size = 13)+ geom_bar(stat="identity", colour="black", size = 0.1)+  scale_fill_manual(values=(
    colour_code9)) + scale_x_continuous(limits = c(0.5,num+0.5), expand = c(0, 0))
#+geom_errorbar(aes(ymin=Rel_ab - sd, ymax=Rel_ab + sd), width = 0.1)

#colour_code9b)[c(1,5,4,3,2)]
```

```
# plot without colour lines
#ggplot(data = x, aes(x=rank,  y= Rel_ab, group = Taxonomy, colour = Taxonomy,
#                    fill =Taxonomy))+ labs(y = "Relative abundance change [%]", x = "")+
#  theme_bw(base_size = 13)+ geom_bar(stat="identity")+  scale_fill_manual(values=colour_code) +
#  scale_x_continuous(limits = c(0.5,100.5), expand = c(0, 0))+  scale_colour_manual(values=colour_code)

# determine what the most abundant classes are
h<-data.frame(rowSums(otu.relative), taxonomy$Class) ; colnames(h)<-c("abund","class")
d<-aggregate(h$abund, by=list(h$class),function(v){sum(v)})
d[order(-d[,2]),] # determine what the most abundant classes are

#############################################################################################
##################
#############################################################################################
##################
#############################################################################################
##################
###### 2) Abundant strains
#plot A - phyogeny of the most abundant strains
colour_code3<- c(rgb(253,160,43,maxColorValue=255), rgb(205,31,13,maxColorValue=255),rgb(72,85,92,maxCol
orValue=255))

# prepare data to read into phyloseq
rownames(otu.relative)<-rownames(otu); rownames(taxonomy)<-rownames(otu); rownames(meta)<- meta[,1]
colnames(otu.relative)<-rownames(meta)
OTU<-otu_table(as.matrix(otu.relative[,which(meta$treatment!="start")]),taxa_are_rows = TRUE)
TAX = tax_table(as.matrix(cbind(taxonomy, rownam = as.character(rownames(taxonomy)))))
SAMPLE<-sample_data(meta[which(meta$treatment!="start"),])

physeq.rel.dens = phyloseq(OTU, TAX,SAMPLE,tree)
physeq.rel.dens.filtered = filter_taxa(physeq.rel.dens, function(x) mean(x) > (0.35), TRUE)
# modify to adjust size
a<-as.data.frame(otu_table(physeq.rel.dens.filtered)) # get ID of taxa
a<-exp(a); for(i in 1:ncol(a)){a[which(a[,i]==1),i]<-0}
a<-otu_table(a,taxa_are_rows = TRUE)
physeq.rel.dens.filtered2 = phyloseq(a, TAX,SAMPLE,tree)

plot_tree(physeq.rel.dens.filtered2, color="treatment", ladderize="left", size="abundance",
        sizebase = 10, base.spacing=0.03,
        label.tips = "Genus",nodelabf=nodeplotblank,plot.margin=0.18, text.size = 2.5)+
  scale_colour_manual(values=colour_code3,aes(x=label.tips)) # you can also set it to label.tips ="rownam" to get I
D

a<-as.data.frame(otu_table(physeq.rel.dens.filtered)) # get ID of taxa
i<-as.data.frame(tax_table(physeq.rel.dens.filtered)) # get ID of taxa

# really messed up taxonomy - would be good to blast search them - ideally the top 100... -> Done!

#############################################################################################
##################
#############################################################################################
##################
#############################################################################################
##################
```

```
###### 2) Abundant strains
#plot C - differences between Classes

# https://www.ncbi.nlm.nih.gov/pmc/articles/PMC5867457/
# https://docs.qiime2.org/2019.10/tutorials/gneiss/

# in the end I decided to go for a nested Wilcox test - I think that makes here more sense than the balances

library(ARTool)

# non-parametric test
p_value<-c()
a<-otu.relative[,which(meta$treatment!="start")]
x<-meta[which(meta$treatment!="start"),]
for (i in 1:nrow(otu.relative)){if(sum(a[i,]>0)){z<-data.frame(as.numeric(a[i,]),x$treatment,x$experiment)
colnames(z)<-c("a","b","c")
m <- art(a ~ b+ (1|c), data=z)
mod1<-anova(m); p_value<-c(p_value, mod1[,5])}else{p_value<-c(p_value,1)}}

# calculations steps (i) aggregate per phylum (ii)

#determine what the most abundant classes are
h<-data.frame(rowMeans(otu.relative[,which(meta$treatment!="start")]), taxonomy$Class) ; colnames(h)<-c("abund
","class")
d<-aggregate(h$abund, by=list(h$class),function(v){sum(v)})
d[order(-d[,2]),] # determine what the most abundant classes are

x<-taxonomy
for(i in 1:nrow(x)){if(x[i,3]!="Alphaproteobacteria"&x[i,3]!="Betaproteobacteria"&x[i,3]!="Gammaproteobacteria"
&
                    x[i,3]!="Deltaproteobacteria"&x[i,3]!="Cytophagia"){x[i,3]<-"others"}}
c1.sum<- aggregate(otu.relative[,which(meta$treatment=="C1")], by = list(x$Class),function(v){sum(v)})
c2.sum<- aggregate(otu.relative[,which(meta$treatment=="C2")], by = list(x$Class),function(v){sum(v)})
a<-data.frame(c1.sum[,1],rowMeans(c1.sum[,-1])-rowMeans(c2.sum[,-1]),
            sqrt(rowSds(as.matrix(c1.sum[,-1]))^2+rowSds(as.matrix(c2.sum[,-1]))^2))
colnames(a)<-c("Class","mean","sd") # which bacteria have benefit from grazing

colour_code9<- c( rgb(51,34,136,maxColorValue=255),rgb(18,119,51,maxColorValue=255),rgb(68,171,154,maxCo
lorValue=255),
                rgb(136,205,238,maxColorValue=255), rgb(220,204,119,maxColorValue=255), rgb(204,101,118,maxCo
lorValue=255),
                rgb(170,68,152,maxColorValue=255), rgb(136,34,84,maxColorValue=255))

min<- c(a$mean-c(0,0,0,0,a$sd[5],0))
max<- c(a$mean+c(a$sd[1:4],0,a$sd[6]))

ggplot(data = a, aes(x=Class, y = mean, group = Class,  fill =Class))+
  theme_bw(base_size = 13) +  geom_bar(stat="identity", colour = "black")+
  geom_errorbar(aes(ymin=min, ymax=max ), width = 0.1)+
  scale_fill_manual(values=(colour_code9))

#now the significant ones
direction<-p_value
direction[which((rowSums(otu.relative[,which(meta$treatment=="C1")])-
                rowSums(otu.relative[,which(meta$treatment=="C2")]))>0)]<-1
```

```
direction[which((rowSums(otu.relative[,which(meta$treatment=="C1")])-
                 rowSums(otu.relative[,which(meta$treatment=="C2")]))==0)]<-0
direction[which((rowSums(otu.relative[,which(meta$treatment=="C1")])-
                 rowSums(otu.relative[,which(meta$treatment=="C2")]))<=0)]<--1

c1.sum_p<- aggregate(otu.relative[which(p_value<0.05&direction>=0),which(meta$treatment=="C1")],
                  by = list(x$Class[which(p_value<0.05&direction>=0)]),function(v){sum(v)})
c1.sum_n<- aggregate(otu.relative[which(p_value<0.05&direction<=0),which(meta$treatment=="C1")],
                  by = list(x$Class[which(p_value<0.05&direction<=0)]),function(v){sum(v)})
c2.sum_p<- aggregate(otu.relative[which(p_value<0.05&direction>=0),which(meta$treatment=="C2")],
                  by = list(x$Class[which(p_value<0.05&direction>=0)]),function(v){sum(v)})
c2.sum_n<- aggregate(otu.relative[which(p_value<0.05&direction<=0),which(meta$treatment=="C2")],
                  by = list(x$Class[which(p_value<0.05&direction<=0)]),function(v){sum(v)})

len_p<-otu.relative[which(p_value<0.05&direction>=0),which(meta$treatment!="start")]
for (i in 1:ncol(len_p)){len_p[which(len_p[,i]>0),i]<-1}
len_p<-rowSums(len_p); len_p[which(len_p>0)]<-1
len_p<- aggregate(len_p,  by = list(x$Class[which(p_value<0.05&direction>=0)]),function(v){sum(v)})
len_n<-otu.relative[which(p_value<0.05&direction<=0),which(meta$treatment!="start")]
for (i in 1:ncol(len_n)){len_n[which(len_n[,i]>0),i]<-1}
len_n<-rowSums(len_n); len_n[which(len_n>0)]<-1
len_n<- aggregate(len_n,  by = list(x$Class[which(p_value<0.05&direction<=0)]),function(v){sum(v)})

# I have to get one row in as it is missing in the negative column
c1.sum_n<-rbind(c1.sum_n[1:2,],c("Cytophagia",rep(0,ncol(c1.sum_p)-1)),c1.sum_n[3:5,])
for(i in 2:ncol(c1.sum_n)){c1.sum_n[,i]<-as.numeric(c1.sum_n[,i])}
c2.sum_n<-rbind(c2.sum_n[1:2,],c("Cytophagia",rep(0,ncol(c2.sum_p)-1)),c2.sum_n[3:5,])
for(i in 2:ncol(c2.sum_n)){c2.sum_n[,i]<-as.numeric(c2.sum_n[,i])}

a<-data.frame(c1.sum[,1],rowMeans(c1.sum_p[,-1])-rowMeans(c2.sum_p[,-1]),
              sqrt(rowSds(as.matrix(c1.sum_p[,-1]))^2+rowSds(as.matrix(c2.sum_p[,-1]))^2),
              rowMeans(c1.sum_n[,-1])-rowMeans(c2.sum_n[,-1]),
              sqrt(rowSds(as.matrix(c1.sum_n[,-1]))^2+rowSds(as.matrix(c2.sum_n[,-1]))^2)   )
colnames(a)<-c("Class","mean_p","sd_p","mean_n","sd_n") # which bacteria have benefit from grazing

cbPalette6 <- c( "#D0B541",  "#7EB875", "#57A2AC", "#4E78C4",  "#CE2220","#E67F33")

ggplot(data = a, aes(x=Class, y = mean_p, group = Class,  fill =Class))+
  theme_bw(base_size = 13) +  geom_bar(stat="identity", colour = "black")+
  geom_errorbar(aes(ymin=mean_p, ymax=mean_p+sd_p), width = 0.1)+
  geom_bar(stat="identity", aes(x=Class, y = mean_n, group = Class,  fill =Class), colour = "black")+
  geom_errorbar(aes(ymin=mean_n-sd_n, ymax=mean_n), width = 0.1)+
  scale_fill_manual(values=(cbPalette6))+scale_y_continuous(limits = c(-82, 22), expand = c(0, 0))

ggplot(data = a, aes(x=Class, y = mean_p*(-1), group = Class,  fill =Class))+
  theme_bw(base_size = 13) +  geom_bar(stat="identity", colour = "black")+
  geom_errorbar(aes(ymin=mean_p*(-1)-sd_p, ymax=mean_p*(-1)), width = 0.1)+
  geom_bar(stat="identity", aes(x=Class, y = mean_n*(-1), group = Class,  fill =Class), colour = "black")+
  geom_errorbar(aes(ymin=mean_n*(-1), ymax=mean_n*(-1)+sd_n), width = 0.1)+
  scale_fill_manual(values=(cbPalette6))+scale_y_continuous(limits = c(-22, 82), expand = c(0, 0))
#Rhodocyclaceae Comamonadaceae Acinetobacter

f<-data.frame(taxonomy,direction, p_value)
f<-subset(f, f$p_value<0.05&f$Genus=="Zymomonas")
```

```
which(taxonomy$Genus=="Zymomonas")


##### new 3B - pie charts

g<- aggregate(rowSums(otu.relative[,which(meta$treatment=="C1")])/length(which(meta$treatment=="C1")),
            by=list(x$Class), function (v) {sum(v)})
h<- aggregate(rowSums(otu.relative[,which(meta$treatment=="C2")])/length(which(meta$treatment=="C1")),
            by=list(x$Class), function (v) {sum(v)})

cbPalette6 <- c( "#D0B541",  "#7EB875", "#57A2AC", "#4E78C4",  "#CE2220","#E67F33")

pie(h$x,labels = "", col=cbPalette6,
   main="Pie Chart of Countries")

##############################################################################################
##################
##############################################################################################
##################
##############################################################################################
##################
###### 3) Abundant strains
#plot A - NMDS plot

rownames(otu.relative)<-rownames(otu); rownames(taxonomy)<-rownames(otu); rownames(meta)<- meta[,1]
colnames(otu.relative)<-rownames(meta)
OTU<-otu_table(as.matrix(otu.relative),taxa_are_rows = TRUE)
TAX = tax_table(as.matrix(cbind(taxonomy, rownam = as.character(rownames(taxonomy)))))
SAMPLE<-sample_data(meta)

physeq.rel.dens = phyloseq(OTU, TAX,SAMPLE,tree)

ord <- ordinate(physeq.rel.dens, method="NMDS", distance="bray") #distance: e.g wunifrac, unifrac, jaccard, bray

plot_ordination(physeq.rel.dens, ord, type = "samples", color = "experiment", shape="treatment")+
  geom_point(size=5,alpha=0.7)+ theme_bw(base_size = 13) +  scale_colour_manual(values=(colour_code9b)[c(1,6,
3,4,5,2,7,8)])

##############################################################################################
##################
#plot B - differences between Classes
# pull together pairwise comparisons for within and between group comparisons
within<-c()
for (i in 1:(nrow(meta)-1)){for (k in (i+1):nrow(meta)){
  if(meta$experiment[i]==meta$experiment[k] & meta$treatment[i]==meta$treatment[k]){a<-c(as.character(meta$e
xperiment[i]),
  as.character(meta$treatment[i]),i,k)
  within<-rbind(within,a)}}}

between<-c()
for (i in 1:(nrow(meta)-1)){for (k in (i+1):nrow(meta)){
  if(meta$experiment[i]==meta$experiment[k] & meta$treatment[i]!=meta$treatment[k] & (meta$treatment[i]=="sta
rt"|
     meta$treatment[k]=="start"))
    {a<-c(as.character(meta$experiment[k]), as.character(meta$treatment[i]),i,k)
```

```
    between<-rbind(between,a)}}}

across<-c()
for (i in 1:(nrow(meta)-1)){for (k in (i+1):nrow(meta)){
  if(meta$treatment[i]==meta$treatment[k])
  {a<-c(as.character(meta$experiment[k]), as.character(meta$treatment[i]),i,k)
  across<-rbind(across,a)}}}

within<-as.data.frame(within); between<-as.data.frame(between); across<-as.data.frame(across)
within[,3]<-as.numeric(as.character(within[,3])); within[,4]<-as.numeric(as.character(within[,4]))
between[,3]<-as.numeric(as.character(between[,3])); between[,4]<-as.numeric(as.character(between[,4]))
across[,3]<-as.numeric(as.character(across[,3])); across[,4]<-as.numeric(as.character(across[,4]))

# now run bray-curtis for all of these pairwise comparisons

numerator<-0; denominator<-0; bray.curtis<-c()
for (r in 1:nrow(within)) {
  for(i in 1:nrow(otu.relative)){
      x<-(abs(otu.relative[i,within[r,3]]-otu.relative[i,within[r,4]]))
      y<-(otu.relative[i,within[r,3]]+otu.relative[i,within[r,4]])
      numerator<-numerator+x
      denominator<-denominator+y}
    bray.curtis<-c(bray.curtis,numerator/denominator)
    numerator<-0; denominator<-0}
within<-data.frame(within,bray.curtis)

numerator<-0; denominator<-0; bray.curtis<-c()
for (r in 1:nrow(between)) {
  for(i in 1:nrow(otu.relative)){
    x<-(abs(otu.relative[i,between[r,3]]-otu.relative[i,between[r,4]]))
    y<-(otu.relative[i,between[r,3]]+otu.relative[i,between[r,4]])
    numerator<-numerator+x
    denominator<-denominator+y}
  bray.curtis<-c(bray.curtis,numerator/denominator)
  numerator<-0; denominator<-0}
between<-cbind(between,bray.curtis)

numerator<-0; denominator<-0; bray.curtis<-c()
for (r in 1:nrow(across)) {
  for(i in 1:nrow(otu.relative)){
    x<-(abs(otu.relative[i,across[r,3]]-otu.relative[i,across[r,4]]))
    y<-(otu.relative[i,across[r,3]]+otu.relative[i,across[r,4]])
    numerator<-numerator+x
    denominator<-denominator+y}
  bray.curtis<-c(bray.curtis,numerator/denominator)
  numerator<-0; denominator<-0
  print(length(bray.curtis))}
across<-data.frame(across,bray.curtis)

# plot the data
colnames(within)<-c("experiment", "treatment", "row1","row2","bray")
colnames(between)<-c("experiment", "treatment", "row1","row2","bray")
colnames(across)<-c("experiment", "treatment", "row1","row2","bray")

# now make means and sd for groups
```

```
w_mean<-aggregate(within$bray, by=list(within$treatment),function(v){mean(v)})
w_sd<-aggregate(within$bray, by=list(within$treatment),function(v){sd(v)})
b_mean<-aggregate(between$bray, by=list(between$treatment),function(v){mean(v)})
b_sd<-aggregate(between$bray, by=list(between$treatment),function(v){sd(v)})
a_mean<-aggregate(across$bray, by=list(across$treatment),function(v){mean(v)})
a_sd<-aggregate(across$bray, by=list(across$treatment),function(v){sd(v)})


colour_code3<- c(rgb(253,160,43,maxColorValue=255), rgb(205,31,13,maxColorValue=255),rgb(72,85,92,maxCol
orValue=255))

ggplot(data = between, aes(x=treatment, y = bray, group = treatment, colour = treatment, fill =treatment))+
  theme_bw(base_size = 13) +  geom_sina(colour = "dark gray")+  geom_violin(trim = FALSE, colour = "black",  al
pha=0.6) +
  stat_summary(    fun.data = "mean_sdl",  fun.args = list(mult = 1),
                 geom = "pointrange", color = "black"  )+  labs(y = "Bray-curtis similarity [within groups]", x = "")+
  scale_fill_manual(values=(colour_code3))

ggplot(data = across, aes(x=treatment, y = 1-bray, group = treatment, colour = treatment, fill =treatment))+
  theme_bw(base_size = 13) +  geom_sina(colour = "dark gray")+  geom_violin(trim = FALSE, colour = "black",  al
pha=0.6) +
  stat_summary(    fun.data = "mean_sdl",  fun.args = list(mult = 1),
                 geom = "pointrange", color = "black"  )+  labs(y = "Bray-curtis similarity [within groups]", x = "")+
  scale_fill_manual(values=(colour_code3)) + scale_y_continuous(limits = c(-0.01, 1.01), expand = c(0, 0))

# stat tests
bartlett.test(across$bray ~ across$treatment) # should be fine in terms of variance homogenity
mod1<-aov(across$bray ~ across$treatment)
summary(mod1)
TukeyHSD(mod1)

bartlett.test(between$bray ~ between$treatment) # should be fine in terms of variance homogenity
mod1<-aov(between$bray ~ between$treatment)
summary(mod1)



# check whether you one has consitently lower number of reads and therefore less taxa -> no!
x<-c()
for (k in 1:ncol(otu)){x<-c(x,sum(otu[,k]))}
a<-cbind(meta,x)
ggplot(data = a, aes(x=experiment, y = x, group = treatment, colour = treatment, shape =treatment,
                          fill =treatment))+
  theme_bw(base_size = 15)+ geom_point(size=1.9)+ scale_shape_manual(values=c(25,22,24))+
  scale_color_manual(values=colour_code)+scale_fill_manual(values=colour_code)

##########################################################################################
#####
```
